# A new ferritin *Sj*Fer0 affecting the growth and development of *Schistosoma japonicum*

**DOI:** 10.1186/s13071-022-05247-1

**Published:** 2022-05-24

**Authors:** Fanyuan Zeng, Cun Yi, Wei Zhang, Shaoyun Cheng, Chengsong Sun, Fang Luo, Zheng Feng, Wei Hu

**Affiliations:** 1 State Key Laboratory of Genetic Engineering, Ministry of Education Key Laboratory of Contemporary Anthropology, Collaborative Innovation Center for Genetics and Development, School of Life Sciences, Fudan University, 2005 Song Hu Road, Shanghai, 200438 People’s Republic of China; 2 Key Laboratory of Parasite and Vector Biology of the Chinese Ministry of Health, WHO Collaborating Center for Tropical Diseases, Joint Research Laboratory of Genetics and Ecology on Parasite-Host Interaction, National Institute of Parasitic Diseases, Chinese Center for Disease Control and Prevention, Shanghai, 200025 People’s Republic of China; 3 State Key Laboratory of Reproductive Regulation and Breeding of Grassland Livestock, School of Life Sciences, Inner Monglia University, Hohhot, 010030 People’s Republic of China

**Keywords:** Ferritin, *Schistosoma japonicum*, Growth and development, RNA interference

## Abstract

**Background:**

Schistosomiasis, an acute and chronic parasitic disease, causes substantial morbidity and mortality in tropical and subtropical regions of the world. Iron is an essential constituent of numerous macromolecules involving in important cellular reactions in virtually all organisms. Trematodes of the genus* Schistosoma* live in iron-rich blood, feed on red blood cells and store abundant iron in vitelline cells. Ferritins are multi-meric proteins that store iron inside cells. Three ferritin isoforms in *Schistosoma japonicum* are known, namely *Sj*Fer0, *Sj*Fer1 and *Sj*Fer2; however, their impact on the growth and development of the parasites  is still unknown. In this study we report on and characterize the ferritins in *S. japonicum.*

**Methods:**

A phylogenetic tree of the *Sj*Fer0, *Sj*Fer1 and *Sj*Fer2 genes was constructed to show the evolutionary relationship among species of genus* Schistosoma*. RNA interference in vivo was used to investigate the impact of *Sj*Fer0 on schistosome growth and development.  Immunofluorescence assay was applied  to localize the expression of the ferritins. RNA-sequencing was performed  to characterize the iron transport profile after RNA interference.

**Results:**

*Sj*Fer0 was found to have low similarity with *Sj*Fer1 and *Sj*Fer2 and contain an additional signal peptide sequence. Phylogenetic analysis revealed that *Sj*Fer0 can only cluster with some ferritins of other trematodes and tapeworms, suggesting that this ferritin branch might be unique to these parasites. RNA interference in vivo showed that *Sj*Fer0 significantly affected the growth and development of schistosomula but did not affect egg production of adult female worms. *Sj*Fer1 and *Sj*Fer2 had no significant impact on growth and development. The immunofluorescence study showed that *Sj*Fer0 was widely expressed in the somatic cells and vitelline glands but not in the testicle or ovary**.** RNA-sequencing indicated  that, in female, the ion transport process and calcium ion binding function were downregulated after *Sj*Fer0 RNA interference. Among the differentially downregulated genes, *Sj*-cpi-2, annexin and insulin-like growth factor-binding protein may be accounted for the suppression of schistosome growth and development.

**Conclusions:**

The results indicate that *Sj*Fer0 affects the growth and development of schistosomula but does not affect egg production of adult female worms. *Sj*Fer0 can rescue the growth of the* fet3fet4* double mutant *Saccharomyces cerevisiae* (strain DEY1453), suggesting being able to promote iron absorption. The RNA interference of *Sj*Fer0 inferred that  the suppression of worm growth and development may via  down-regulating *Sj*-cpi-2, annexin, and IGFBP.

**Graphic Abstract:**

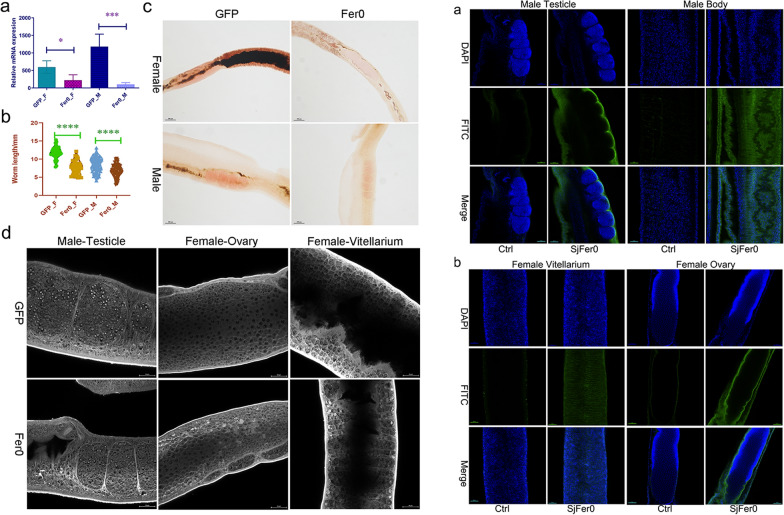

**Supplementary Information:**

The online version contains supplementary material available at 10.1186/s13071-022-05247-1.

## Background

Iron is is an essential constituent of numerous macromolecules participating in many biological reactions and processes that sustain life. However, iron overload is harmful and causes Fenton's response, resulting in the production of cytotoxic oxygen free radicals [1] and, sometimes, diseases, such as, neurodegeneration with brain iron accumulation (NBIA) [2]. Thus, the maintenance of iron homeostasis in organisms is crucial . 

Food is the only source of iron for mammals. The absorbed iron crosses the intestine basolateral membrane with the aid of iron transporter ferroportin (FPN) and passes into the bloodstream where it is oxidized by multicopper oxidase hephaestin (HEPH) [3, 4] to ferric ion (Fe^3+^). When Fe^3+^ in the blood encounters transferrin (Tf), which contains two high-affinity Fe sites, the Tf-Fe2 complex is formed. Tf-Fe2  is then absorbed by transferrin receptor 1 (Tfr1)-expressing cells (e.g. erythrocytes, liver cells) through Tfr1-mediated endocytosis [5]. The endocytosed Tf-Fe2/TFR1 complex releases Fe^3+^, which is then reduced to ferrous ion by Steap3 (six-transmembrane epithelial antigen of prostate 3) [6] from Tf in the early acidic environment, and further transported into the cytoplasm by divalent metal transporter 1 (DMT1) [7, 8].

Experiments in cardiomyocytes [9] and dopaminergic neurons [10] have shown that Tfr1 plays a vital role in iron uptake, while most non-erythroid cells can absorb iron without Tfr1. When the iron concentration exceeds the binding capacity of Tf, non-transferrin-bound iron (NTBI) will appear, of which ferritin is a primary carrier. Ferritin is a spherical protein complex formed by 24 subunits of heavy-chain (H-chain) and light-chain (L-chain) ferritin that is able to store up to 4000 iron atoms [11], although the sequences from different species clearly vary. T cell immunoglobulin-domain and mucin-domain (TIM) proteins comprise a receptor family and play important roles in immunity with broader functions. Studies have shown that TIM-2 can mediate the endocytosis of H-ferritin (FTH1) in spleen B cells [12], while Scavenger receptor member 5 (Scara5) can facilitate the endocytosis of L-ferritin in kidney cells [13]. Moreover, Zip14, a member of the SLC39A zinc transporter family, can mediate NTBI uptake into cells [14].

The cytoplasmic labile iron pool supplies iron to the mitochondrion for controlling numerous metabolic reactions. Iron trafficking has two destinations: cytosol or mitochondria. Various iron-dependent proteins in the cytosol function in iron metabolism and utilization. Human poly (rC) binding protein 1 (PCBP1) is an iron chaperone that binds iron and delivers it to ferritins, the cytosolic iron storage proteins [15, 16]. However, intracellular iron is also transported to mitochondria via the complex of mitoferrin and ATP-binding cassette sub-family B member 10 Abcb10) for the synthesis of heme and other iron-containing proteins [17, 18]. It was demonstrated that H-ferritin, with ferric oxidase activity, mineralizes Fe^2+^, while L-ferritin promotes the transfer of electrons across polymer nanocages in this redox process [19].

Schistosomiasis, a parasitic disease, is caused by flat worms of the genus* Schistosoma*, posing substantial morbidity and mortality in tropical and subtropical regions worldwide. It is considered by WHO to be one of the most prevalent neglected tropical diseases, second only to malaria [20]. Mature adult worms reside in the mesenteric (*Schistosoma mansoni* and *Schistosoma japonicum*) or pelvic (*Schistosoma haematobium*) veins, where female worms lay eggs that are subsequently excreted in the stool or urine. Schistosomes feed on red blood cells (RBCs). The host's blood contains a large amount of iron, including heme iron, transferrin-bound iron (Tf-Fe2) and NTBI (e.g. ferritin mineralized iron and organic acid complex iron). Culture experiments of *S. mansoni* in vitro showed that both host Tf-Fe2 and free Fe^3+^ could promote the development of schistosomula [21], suggesting that iron is closely related to the development of schistosomes. Female schistosome tissues are rich in iron [27] and thus iron acquisition is integral for the growth and development of schistosomula. The ferrous iron transporter DMT1 in *S. mansoni* was shown to transport iron and found mainly located in the tegument [22]. A cytochrome identified in *S. japonicum*, *Sj*cytb561, is a member of the cytochrome b561 (cyts-b561) family of ascorbate-reducing transmembrane proteins and shows iron reductase activity. It is also mainly expressed in the tegument [23], implying that *Sj*cytb561 facilitates iron transportation by DMT1 [24]. In addition, two isoforms of the iron storage protein ferritin, Fer1 and Fer2, have been identified in* S. mansoni*. Fer2 is called somal ferritin and preferentially expressed in males, while Fer1 is called yolk ferritin and is present in the yolk platelets of vitelline cells [25, 26]. Fer1 and Fer2 are also found in *S. japonicum*, and the Fer1 (yolk ferritin) is likely transcribed by vitelline cells and the iron stored in ferritin is incorporated into eggshells [27]. However, the function of ferritin and the impact on the growth and development of schistosomes remains unclear.

In this paper we describe the identification and molecular characterization of a new ferritin isoform, *Sj*Fer0, in *S. japonicum* (Genbank ID: ACE06912.1). To investigate the function of the ferritin family, we compared the three forms of *S. japonicum* ferritins (*Sj*Fer0, *Sj*Fer1 and *Sj*Fer2) and explored their possible impact on schistosome growth and development.

## Methods

### Animals and parasites

*Schistosoma japonicum* cercariae (Anhui province isolate) were provided by the Department of Vector Biology, National Institute of Parasitic Disease, Chinese Center for Disease Control and Prevention (NIPD, China CDC). C57BL/6 mice (8 weeks age), purchased from Shanghai Animal Center, Chinese Academy of Sciences (Shanghai, China), were infected with *S. japonicum* cercariae. Two specific pathogen-free Japanese White Rabbits were each injected with 0.25 mg recombinant *Sj*Fer0 protein 4 times every 2 weeks and then sacrificed at week 10; polyclonal antibodies were extracted from the collected blood samples by ABclonal Technology Co., Ltd (Woburn, MA, USA).

All animal experiments complied with the Guide for the Care and Use of Laboratory Animals and were approved by the Ethics Committee of the National Institute of Parasitic Diseases, Chinese CDC (Shanghai, China).

### Bioinformatics of three *S. japonicum* ferritin isoforms

Information on the sequences of *Sj*Fer0, *Sj*Fer1 and *Sj*Fer2 is available on the Uniprot website (https://www.uniprot.org/) under accession numbers B3GUY2, C1LRQ1 and C1L7G5, respectively. Protein domains were analyzed by the SMART tool (http://smart.embl-heidelberg.de/) [28]. Information on the chromosomal position of the three three ferritins is derived from the V3 genome assembly *S. japonicum* (SRA accession number PRJNA739049).

### Phylogenetic analysis of Ferritin protein

Ferritin protein sequences of multiple species were acquired from the Uniprot database. MEGA-X software was used to construct the phylogenetic tree with the maximum likelihood method, then improved by the iTOL online tool (https://itol.embl.de/index.shtml).

### Examination of the expression profile of *Sj*Fer0, *Sj*Fer1 and *Sj*Fer2 at different time points after infection

C57BL/6 mice were infected with 60 cercariae percutaneously through the shaved abdomen. Mice were euthanized at two-day interval during the  14-30  days post-infection (dpi), respectively, and perfused with a 4 °C saline solution containing heparin sodium via the hepatic portal vein to obtain schistosomula or adult schistosomes. Male and female *S. japonicum* collected at each time point, with the exception of 14 dpi, were separated with a soft brush for total RNA extraction with RNAiso Plus (TRIzol; Takara Bio, Shiga, Japan). The expression profile of ferritin messenger RNAs (mRNAs) was examined by quantitative PCR (qPCR) with the PrimeScript RT reagent Kit with gDNA Eraser (Perfect Real Time; Takara Bio) and 2× SYBR Green qPCR Master Mix (Bimake, Houston, TX, USA). The qPCR primers of *Sj*Fer0, *Sj*Fer1, *Sj*Fer2 and* 26S* proteasome non-ATPase regulatory subunit 4 (PSMD4) for internal reference [29] were designed with the Primer Premier 6.0 software package (Additional file 1: Table S1).

### RNA interference assay in vivo

RNA interference (RNAi) was performed using the method as described previously [30]. To produce double-stranded RNA (dsRNA) for the assay, the target DNA sequences were amplified from* S. japonicum* complementary DNA (cDNA) by primers (Additional file 2: Table S2) containing the T7 promoter and then transcribed to dsRNA by the MEGAscriptTM T7 High Yield Transcription kit (Invitrogen, Thermo Fisher Scientific, Waltham, MA, USA). The mice were  assigned to  *Sj*Fer0, *Sj*Fer1, *Sj*Fer2 and green fluorescent protein (GFP) groups, with four mice in each group, and each group was treated with 10 μg target dsRNA via intravenous injection into the tail at 1, 6, 10, 14, 18, 22 and 26 dpi. Schistosomes were recovered by perfusion [30] at 30 dpi. The worms were grouped  with six pairs each, washed three times with diethyl pyrocarbonate (DEPC)-treated phosphate buffered saline (PBS), frozen in liquid nitrogen and then stored until RNA extraction.

To assess longer time of interference up to 42 days on the impact of *Sj*Fer0 on schistosome growth and development or egg production and hepatic fibrosis, 16 mice, in four groups of four mice each, respectively, were administered 10 μg *Sj*Fer0 dsRNA, or GFP dsRNA as a control, under two injection schemes: scheme A,  injections on 1, 6, 10, 14, 18, 22, 26, 30, 34 and 38 dpi; scheme B, injections on 26, 30, 34 and 38 dpi. The mice were sacrificed to recover schistosomes at 42 dpi, and the subsequent treatment of the worms was the same as described for the 30 dpi assay.

### Measuring schistosome body length and carmine alum staining

Schistosomes were separated and treated with AFA buffer (alcohol-formalin-acetic acid: alcohol 95%, formalin 3%, glacial acetic acid 2%). Digital images of the schistosomes were taken, and the images analyzed with ImageJ software(https://imagej.nih.gov/ij/) to measure schistosome body length.

The schistosomes were stained with Mayer's carmine alum stain after dehydration through an alcohol gradient (30, 50 and 70% ethanol). The worms were then permeabilized with methyl salicylate after decolorization with hydrochloric acid alcohol (3% hydrochloric acid, 70% ethanol) and dehydration through an alcohol gradient (70, 85 and 100% ethanol). As a final step, the parasites were mounted on a Nikon fluorescence microscope and Nikon A1-Ni laser confocal microscope (Nikon Corp., Tokyo, Japan) for morphological observation.

### Hematoxylin/eosin staining of liver tissue and egg counting in liver

A portion of the mouse liver at 42 dpi was fixed with formaldehyde solution and sent to Wuhan Servicebio Co., Ltd (Wuhan, China) for staining with hematoxylin/eosin (HE). The remaining liver tissues were weighed (*W*, in grams) and digested with 5% NaOH (w/v) (*V*, in milliliters) at 37 °C for 24 h for egg counting under microscope (10 μl of digested suspension).  The eggs in each sample were counted at least three times and a mean egg number (*x*) determined. The number of paired schistosomes was recorded (*n* pairs) when recovering. Consequently, the number of eggs that each pair of schistosomes produced per gram of liver tissue (*N*) is given by the following formula: *N* = 100 × *x* × *V*/(*n* × *W*).

### Preparation of a rabbit polyclonal antibodies against *Sj*Fer0

The *Sj*Fer0 sequence was amplified with the forward primer 5ʹ-AATGGGTCGCGGATCCCCTATTCAAACTAACGGTGAG-3ʹ and reverse primer 5ʹ-AATGGGTCGCGGATCCCCTATTCAAACTAACGGTGAG-3ʹ, subsequently cloned into the pET-28a vector and then linearized by BamHI and XhoI enzymes (New England Biolabs [NEB], Ipswich, MA, USA) for expression in *Escherichia coli* BL21 (DE3) (TIANGEN BIOTECH, Beijing, China). The expressed recombinant *Sj*Fer0 was purified by Ni column chromatography and concentrated by centrifugation at 5000 *g* at 4 °C in an ultrafiltration tube (MilliporeSigma, Burlington, MA, USA). The purified and concentrated recombinant *Sj*Fer0 protein was used to produce rabbit polyclonal antibody against *Sj*Fer0 by ABclonal Technology Co., Ltd (check section 3 for details).

### Immunofluorescence localization of *Sj*Fer0

Schistosomes recovered at 35 dpi from infected mice were cultured in Dulbecco’s modified Eagle medium (DMEM) with 10% fetal bovine serum (FBS) at 37 °C until the paired parasites separated. The worms were then killed by 0.6 M MgCl_2_ and fixed in 4% paraformaldehyde for 4 h at room temperature. The fixed samples were washed 10 min in PBSTx (PBS, 0.3% Triton X-100), 50% methanol (prepared with PBSTx) and 100% methanol. Samples could be stored in methanol at – 20 °C for several weeks.

The immunofluorescence assay was performed as previously described [31] with slight modifications. Briefly, on day 1, worm samples were hydrated for 10 min in 50% methanol (prepared with PBSTx) and PBSTx, then bleached for 90 min under intense light in a bleaching buffer (900 ul H_2_O, 50 ul formamide, 25 μl 20× SSC, 40 μl 30% H_2_O_2_), followed by two 1-min washes in PBST (PBS, 0.2% Tween20), digestion for 30 min in proteinase K (2 μg/ml) and 10 min in 4% paraformaldehyde (PFA), then 2% PFA and a wash in PBST. As a last step, the worm samples were blocked in blocking buffer (5% serum prepared with PBST) at 4 °C overnight. On day 2, three 20-min washes in PBST were carried out. Then the females were treated with 50 mM CuSO_4_ (pH = 5.0, regulated with 1 M NH_4_Ac) for 6 h and the males were treated with 0.5% Sudan black B for 6 h, followed by six 20-min washes in PBST. Next, the parasites were incubated in *Sj*Fer0 antibody or the negative rabbit-derived serum solution (1:500 dilutions in blocking buffer) at 4 °C overnight.  On day 3, five 20-min washes in PBST were carried out. Then the commercialized secondary antibody Goat Anti-Rabbit IgG H&L (Alexa Fluor 488; ab150077; 1:1000 dilution; Abcam, Cambridge, UK) was applied, and the solution was incubated at room temperature for 3 h, followed by three 20-min washes in PBST. The 4’,6-diamidino-2-phenylindole (DAPI) dye (Thermo Fisher Scientific, Waltham, MA, USA) was applied at 1:10,000 dilution and incubated at 4 °C overnight. On the last day, the worms were washed three times in PBST (20 min each wash) and then transferred to 80% glycerin for mounting and observation under a laser confocal microscope (Nikon Corp.).

### Functional expression of *Sj*Fer0 in *Saccharomyces cerevisiae*

The *fet3fet4* double mutant *Saccharomyces cerevisiae* (strain DEY1453) is defective in low-affinity and high-affinity iron transport systems [32]**. **It shows more insufficient growth than the wild-type strain DY1450 on an iron-limited medium. These strains were kindly provided by Dr. Chen R (Chinese Academy of Agricultural Sciences, Beijing, China) with permission from Dr. David Eide (Nutritional Science Program, University of Missouri, Columbia, MO, USA).

The protocol used in the present study was a slight modification of that described by Ballesteros et al. [33]. Briefly, the *Sj*Fer0 sequence was first amplified with forward primer 5′-CCGAGCTCGGATCCATGAAAATCATGATGTTGATGAC-3′ and reverse primer 5′-GATGCATGCTCGAGTCAACGAAGTTCTTTATCCATC-3′ and then cloned into the pYES2 vector linearized by the BamHI and XhoI enzymes. Empty pYES2 vector was transformed with PEG and LiAc into wild-type strain DY1450 and* fet3fet4* double mutant strain DEY1453 as a positive and negative control, respectively. The pYES2-*Sj*Fer0 vector was transformed into strain DEY1453. Culture at 30 °C for several days on uracil (URA)-selective synthetic defined (SD) media (Invitrogen, Thermo Fisher Scientific) supplemented with adenine hemisulfate and 2% glucose was used to select the pYES2-containing transformants because pYES2 expresses the* URA3* gene.

A single colony was inoculated into URA-selective SD liquid culture media at 30 °C overnight. The cultures were then diluted in series (OD_600_ = 1.0, 0.1, 0.01 and 0.001), following which 5 μl of each dilution was spotted onto selective SD medium URA− plates supplemented with adenine hemisulfate and 2% galactose (for induced expression), 1% raffinose and 10 μM ferric chloride. The plates were incubated at 30 °C to observe colony’s growth.

### RNA-sequencing after *Sj*Fer0 dsRNA interference in vivo

C57BL/6 mice infected with 60 cercariae were injected with 10 μg *Sj*Fer0 dsRNA or GFP dsRNA on 14 dpi and 18 dpi. The mice were sacrificed at 22 dpi and schistosome samples were collected. Second-generation RNA sequencing (RNA-Seq) was performed by Beijing Novogene Technology Co., Ltd. (Novogene, Beijing, China). Quality control (QC) of the raw RNA-Seq data was performed using the FASTQC program. Low-quality reads and adapter contamination were trimmed using the fastp tool [34]**.**

The STAR tool [35] was used to map the clean reads to V3 genome assembly *S. japonicum* (SRA accession number: PRJNA739049). Transcript abundances were imported into the R program after further estimating with RSEM [36]. Differential expression analysis was performed with R package DEseq2, and Gene Ontology (GO) enrichment analysis was performed with the R package clusterProfile [37].

### Statistical analysis

Digital experimental data were visualized by GraphPad Prism 8.0 software (GraphPad Software, San Diego, CA, USA). The results were analyzed using t-test. Results were considered to be significantly different at *P < 0.05, **P < 0.01, ***P < 0.001 and ****P < 0.0001.

## Results

### Protein domain and chromosome locations of ***S. japonicum*** ferritin

**Fig. 1:**
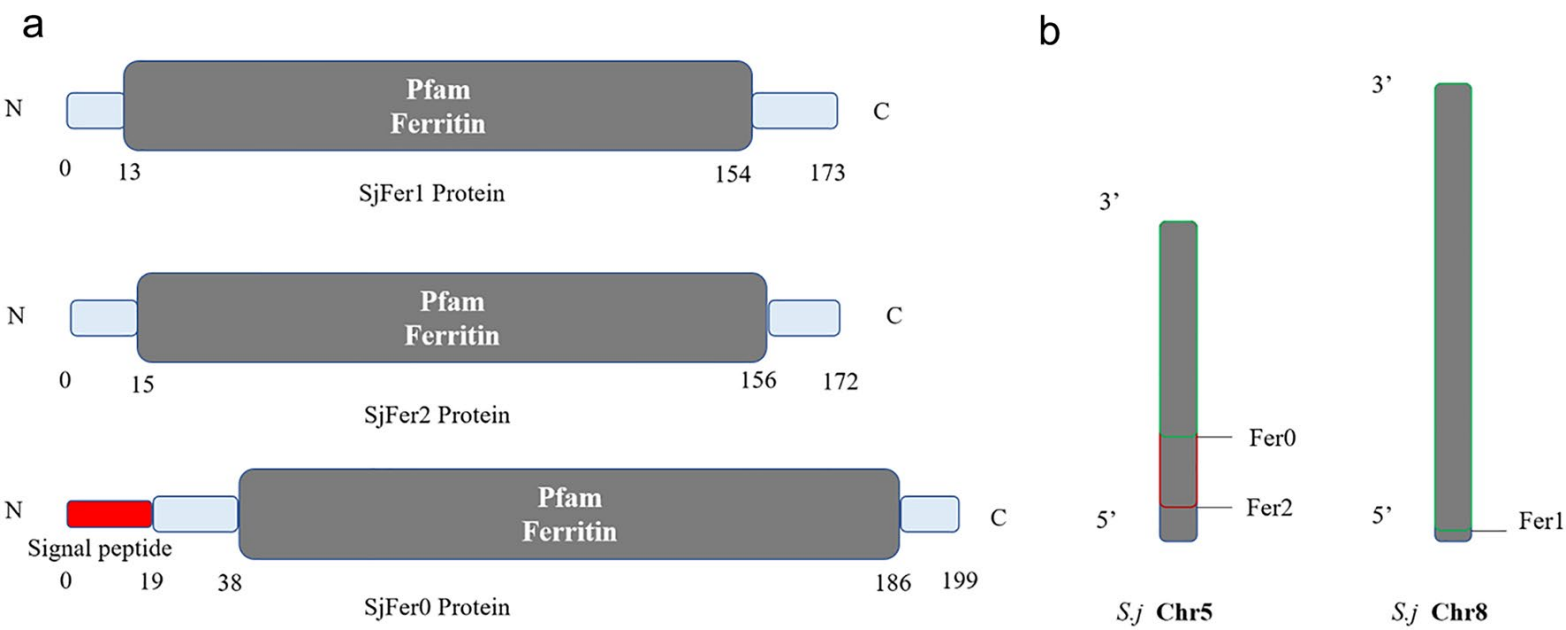
Domain and chromosome locations of the three *Schistosoma japonicum* ferritins (*Sj*Fer0, *Sj*Fer1 and *Sj*Fer2). **a** Protein domain of *S. japonicum* ferritins. *Sj*Fer0 contains an additional signal peptide sequence in addition to a conserved domain. **b** Location on chromosomes of *S. japonicum* ferritins. *Sj*Fer0 and *Sj*Fer2 are located at a distant from each other on chromosome 5, and *Sj*Fer1 is on chromosome 8

*Sj*Fer0 has a low similarity with *Sj*Fer1 and *Sj*Fer2 (Protein identity: 30 ~ 40%), and little is known about its biological traits. An online tool (SMART [Simple Modular Architecture Research Tool]; http://smart.embl-heidelberg.de/) [28] was used to analyze the protein domain. The results showed that, in addition to a conserved ferritin domain, *Sj*Fer0 contains an additional signal peptide sequence, suggesting that it may be secreted extracellularly (Fig. 1a). Based on the V3 genome assembly *S. japonicum* (SRA accession number: PRJNA739049), *Sj*Fer0 and *Sj*Fer2 are located at distant locations on chromosome 5, while *Sj*Fer1 is located on chromosome 8 (Fig. 1b).

### Phylogenetic analysis of ferritins

**Fig. 2:**
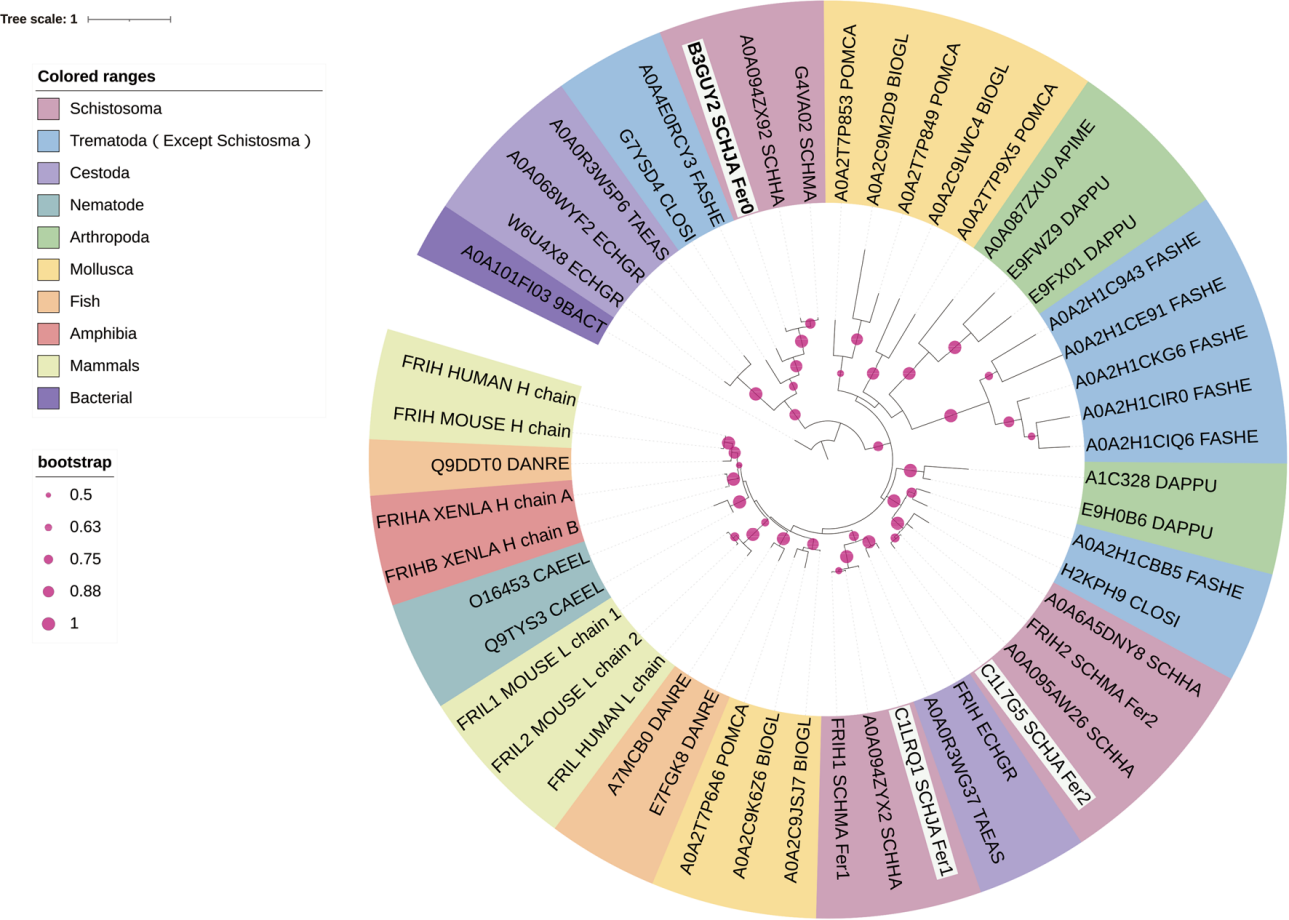
Phylogenetic analysis of ferritins. The sequence ID consists of the Uniprot entry name + species name + protein annotation. Protein sequences were divided into ten groups that are color-coded (see list to the top left of figure): Schistosome (*Schistosoma japonicum* [SCHJA], *S. mansoni* [SCHMA] and *S. haematobium* [SCHHA]); Other trematodes (*Clonorchis sinensis* ([lOSI], *Fasciola hepatica* [FASHE]; Cestoda (*Taenia asiatica* [TAEAS], *Echinococcus granulosus* [ECHGR]); Nematodes *(Caenorhabditis elegans* [CAEEL]); Mollusca (*Pomacea canaliculata* [POMCA], *Biomphalaria glabrata* [BIOGL]); Arthropods (*Oncopeltus fasciatus* [ONCFA], *Apis mellifera* [APIME] and *Daphnia pulex* [DAPPU]); Fish (*Danio rerio* [DANRE]; Amphibians (*Xenopus laevis* [XENLA]; Mammals (*Homo sapiens* ([HUMAN], *Mus* [MOUSE]); Bacteria (*Thermodesulfobacterium Commune* ([BACT])

Ferritin proteins in other organism species acquired from the Uniprot database were used to construct the phylogenetic tree (Fig. 2). *Sj*Fer0, *Sj*Fer1 and *Sj*Fer2 were located into different clades (designated with white background). *Sj*Fer1 and *Sj*Fer2 had a shorter evolutionary distance with mammal ferritin than *Sj*Fer0. Each species has two or more isoforms of ferritin. Among the compared species, only certain ferritins of trematodes and tapeworms were found to cluster with *Sj*Fer0, suggesting that this ferritin branch might be unique to these parasites.

### Expression patterns of *Sj*Fer0, *Sj*Fer1 and *Sj*Fer2 at different time points after infection

**Fig. 3:**
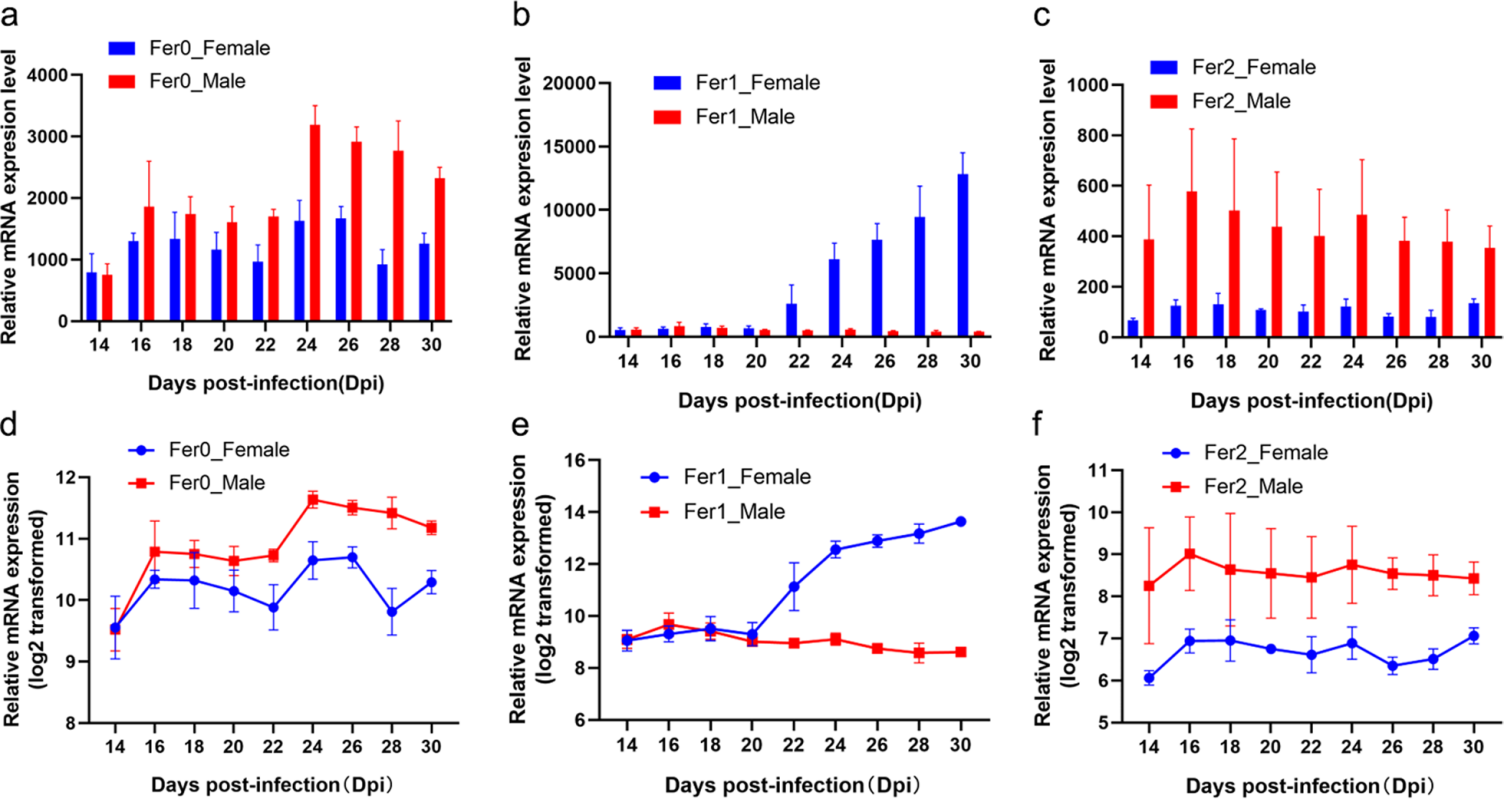
Expression patterns of *Sj*Fer0, *Sj*Fer1 and *Sj*Fer2 at different time points after infection. **a**–**f** mRNA expression of *Sj*Fer0, *Sj*Fer1 and *Sj*Fer2 at 14 ~ 30 dpi (**a**–**c**) and 14 ~ 30dpi (**d**–**f**). Each sample had four duplicates. Error bars: 95% confidence intervals

To further compare and understand the characteristics of the three ferritins of *S. japonicum*, the parasites were recovered from infected mice at different time points after infection to examine the expression profiles of the ferritins. The expression levels of *Sj*Fer0, *Sj*Fer1 and *Sj*Fer2 in female and male worms were determined by reverse transcription-qPCR. The expression level of *Sj*Fer0 mRNA showed an overall upward trend, with male worms having a higher expression level than females (Fig. 3a, d). The expression level of yolk gland ferritin *Sj*Fer1 was relatively stable in males but increased sharply after 20 dpi in females (Fig. 3b, e), which corresponds with the rapid development of female vitellarium after 20 dpi [38]. The expression level of somal ferritin *Sj*Fer2 fluctuated modestly, with male worms found to have about twice the expression level of female worms (Fig. 3c, f).

### *Sj*Fer0 affects the growth and development of schistosomes

**Fig. 4:**
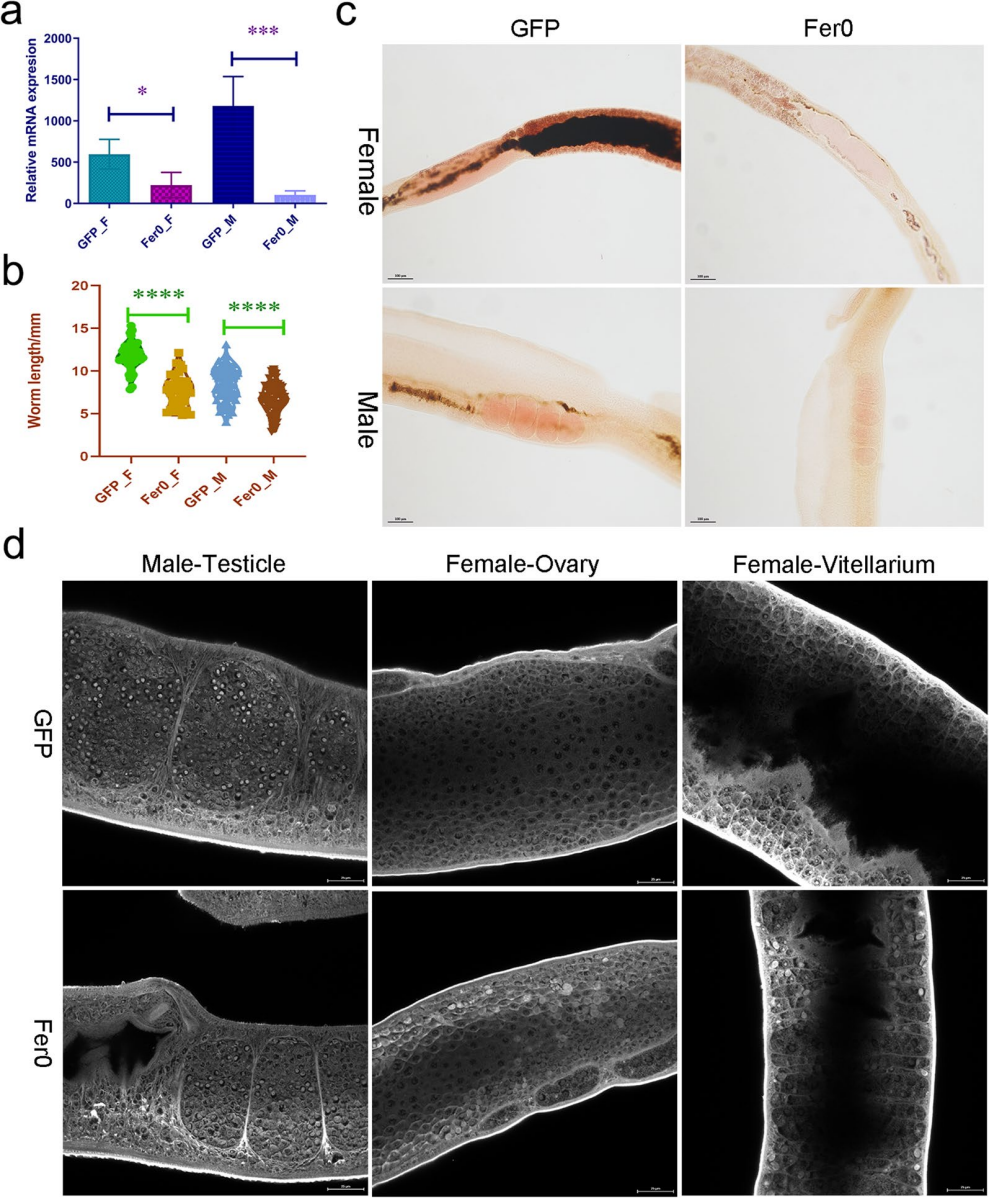
*Sj*Fer0 dsRNA interference in vivo*.* **a** *Sj*Fer0 mRNA expression levels detected by RT-qPCR. Error bars: 95% confidence intervals, *n* = 4. Asterisks indicate a significant difference at *P < 0.05, ***P < 0.001 (t-test). **b** Worm body length measurements. Asterisks indicate a significant difference at ****P < 0.0001 (t-test, *n* > 30). **c** Schistosome stained with carmine alum was observed by fluorescence microscopy. The gonad was less stained and there was little hemozoin deposition in the *Sj*Fer0 dsRNA interference group. Scale bar: 100 um. **d** Schistosome stained with carmine alum observed by laser scanning confocal microscopy (LSCM). The *Sj*Fer0 dsRNA interfered worm has a fewer eggs and less yolk than the GFP group. Scale bar: 25 um. Abbreviations: F, Female; GFP, green fluorescent protein; M, male; RT–qPCR, reverse transcription–quantitative PCR

A dsRNA interference study revealed that mRNA expression levels of the worms injected with *Sj*Fer0 dsRNA, as detected by RT-qPCR, fell significantly in both female and male worms (Fig. 4a). It is noteworthy that the body lengths of both female and male worms were also significantly reduced (Fig. 4b), suggesting *Sj*Fer0 affects schistosome growth. Morphological observation following staining with carmine alum showed lower staining of gonads and little hemozoin deposition, indicating suppression of development of female ovary, female vitellarium and male testicle (Fig. 4c, d).

For the *Sj*Fer1 group, the *Sj*Fer1 mRNA expression level in male worms decreased significantly in comparison with the control, but *Sj*Fer1 expression level in the female worms did not change signficantly from the control (Additional file 3: Fig. S1a). Also, the *Sj*Fer1 dsRNA-interfered worms presented no changes in growth and development (Additional file 3: Fig. S1b-d).

In the *Sj*Fer2 group, the *Sj*Fer2 mRNA expression level in both male and female worms was significantly knocked down (Additional file 4: Fig. S2a). However, only the body length of male worms was reduced (Additional file 4: Fig. S2b). Little effect was observed in the female ovary and vitellarium (Additional file 4: Fig. S2c-d).

### SjFer0 affects the schistosomula rather than adult schistosomes

**Fig. 5:**
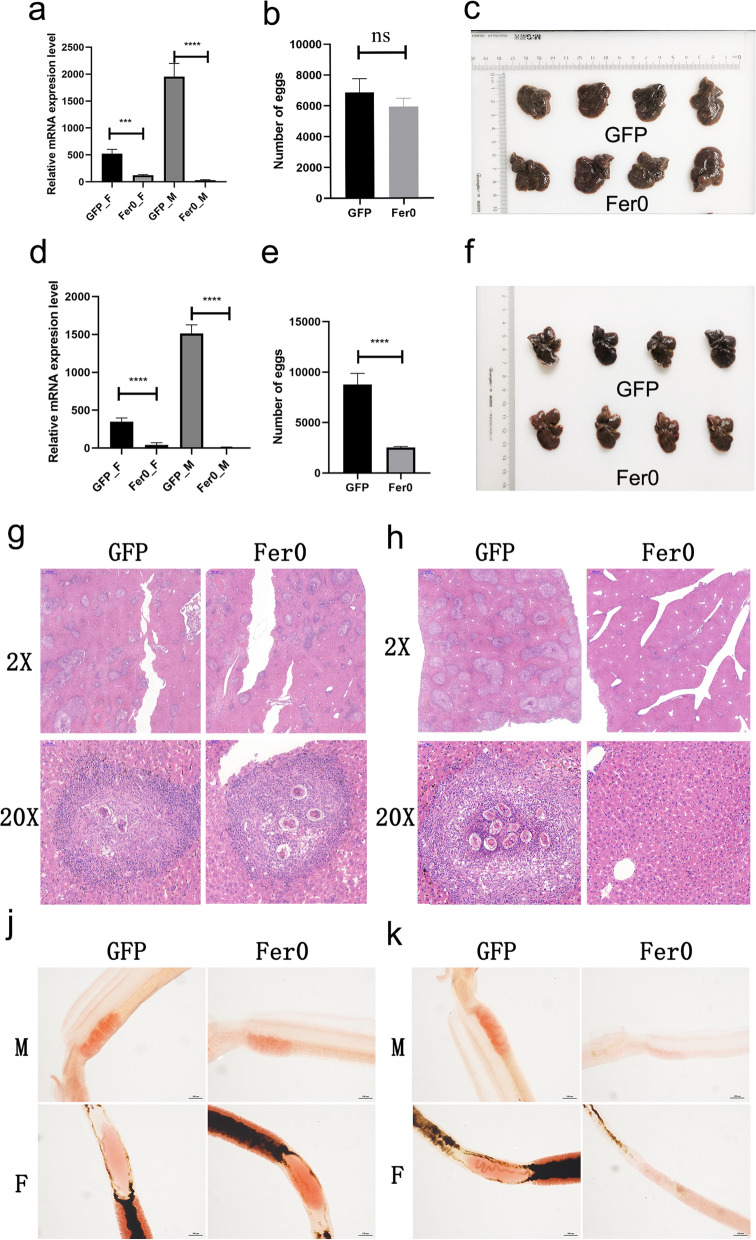
*Sj*Fer0 functions compared between schistosomula and adult schistosomes. **a**–**c**, **g**, **j** dsRNA injection was started on 26 dpi. **d**-**f**, **h**, **k** dsRNA injection was started on 1 dpi. **a**, **d** *Sj*Fer0 mRNA expression levels detected by RT-qPCR. Error bars: 95% confidence intervals, *n* = 4. Asterisks indicate significant difference at ***P < 0.001 and ****P < 0.0001 (t-test). **b**, **e** Effect of dsRNA interference on the egg production of schistosomes. The statistics are based on the egg number of each pair of schistosomes in 1 g mouse liver tissue. Error bars: 95% confidence intervals, *n* = 4. Asterisks indicate signifiant difference at ****P < 0.0001; ns indicates no significant difference (*P* > 0.05) (t-test). **c**, **f** Excised mouse liver. **g**, **h** HE staining of liver tissues. **j**, **k** Schistosome stained with carmine alum. Observation by fluorescence microscopy. Scale bar: 100 um

Two dsRNA injection schemes were carried out to explore whether SjFer0 affects adult schistosome egg production. The dsRNA interference was carried out at 1-26 dpi in scheme A, and at 26-42 dpi in scheme B, respectively. Although the SjFer0 mRNA expression of both female and male worms was knocked down significantly in scheme B (Fig. 5a), no noticeable impact was found on egg production (Fig. 5b). Furthermore, there was no distinction in the liver fibrosis in appearance (Fig. 5c) and HE staining (Fig. 5g) between interference and control group mice. Lastly, the SjFer0 dsRNA knocked-down worms stained with carmine showed no impact on worm development (Fig. 5j).

Comparatively, in scheme A, the ability of egg production was significantly reduced (Fig. 5e), and liver fibrosis was alleviated (Fig. 5f, Fig. 5h) when SjFer0 mRNA expression was knocked down (Fig. 5d). Besides, parasites' development was retarded (Fig. 5k).

In summary, SjFer0 interference at the schistosomula stage affects the growth of schistosomula and eventually leads to a reduction of egg production, while the interference at the adult stage, did not result in a reduction of egg production.

### *Sj*Fer0 was widely expressed in the somatic cells and vitelline glands but not in the testicle or ovary

**Fig. 6:**
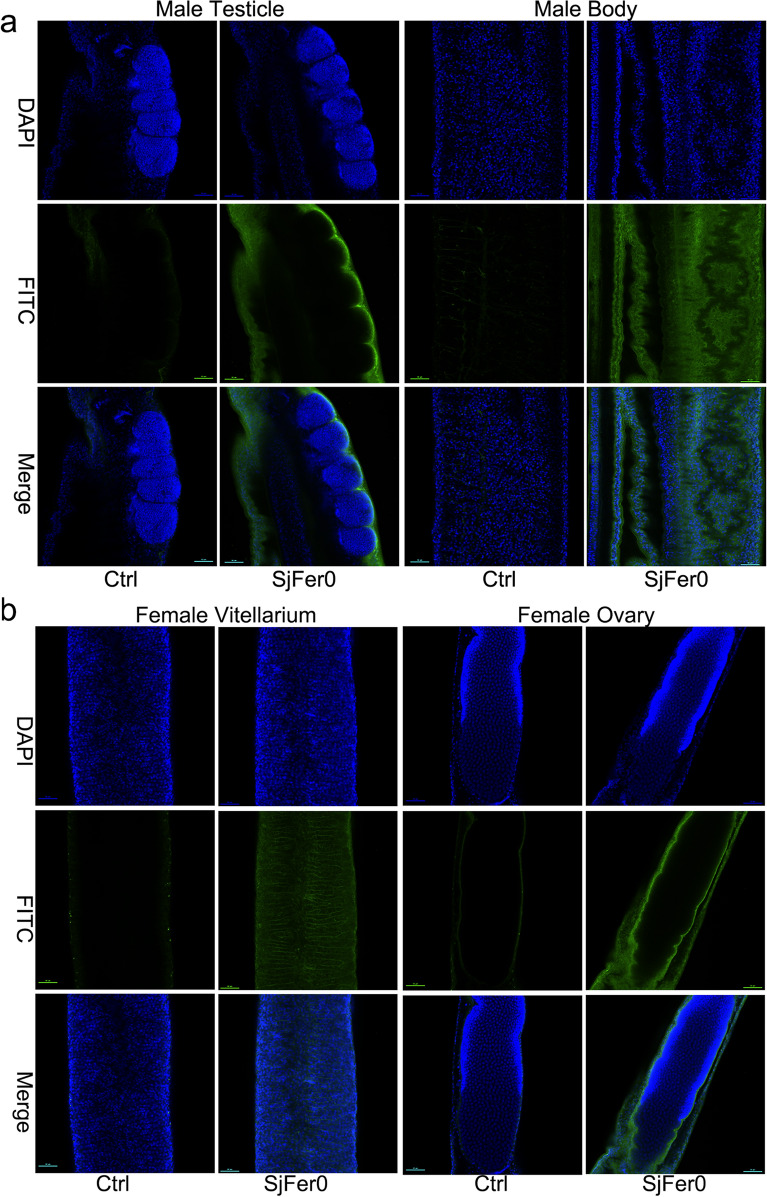
*Sj*Fer0 immunofluorescence. **a** *Sj*Fer0 immunofluorescence in male worms. *Sj*Fer0 protein is widely expressed in the somatic cells of adult males but not in the testicle. **b** *Sj*Fer0 immunofluorescence in females. *Sj*Fer0 is detected in the somatic cells and vitelline glands of adult females but not in the ovaries. Vitelline gland expression is extracellular. The experimental mice group (*Sj*Fer0) is that injected with *Sj*Fer0 polyclonal antibody, and the Control group (Ctrl) is rabbit-derived negative serum. The secondary antibody was FITC-labeled Goat Anti-Rabbit IgG H&L (Alexa Fluor 488). Abbreviations: DAPI (4’,6-diamidin-2-phenylindol): blue staining; FITC (fluorescein isothyocianate): green staining. Scale bar: 50 um

Recombinant *Sj*Fer0 protein was expressed, purified and injected into rabbits to obtain a rabbit polyclonal antibody against *Sj*Fer0, which was then used for the immunofluorescence assay. The specificity of the polyclonal antibodies was tested by western blot before the immunofluorescence assay (Additional file 5: Fig. S3). *Sj*Fer0 protein was found to be widely expressed in the somatic cells of adult males but not in the testicle (Fig. 6a). Likewise, *Sj*Fer0 protein was detected in somatic cells and vitelline glands of adult females but not in the ovary (Fig. 6b). Interestingly, vitelline gland expression was observed to be extracellular (Fig. 6b).

### Recombinant *Sj*Fer0 promotes the growth of the* fet3fet4S. cerevisiae* double mutant

The yeast *S. cerevisiae* has been used as a model organism to study the regulation of iron uptake, recycling and mobilization. We used the method described by Ballesteros et al. [33] and constructed *Sj*Fer0 into the i*fet3fet4* double mutant *S. cerevisiae* (strain DEY1453) [39] to explore the function of *Sj*Fer0.

**Fig. 7:**
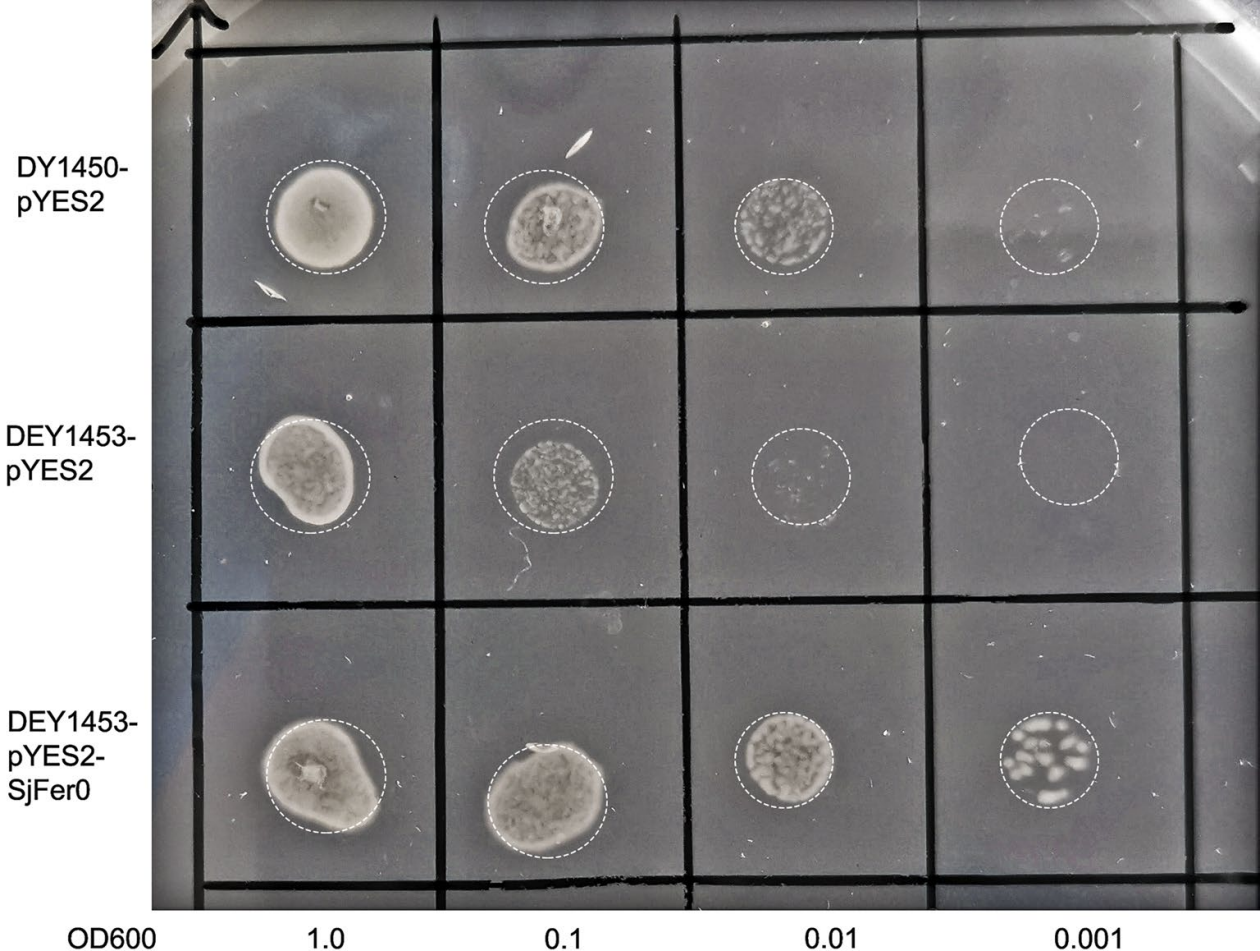
Functional expression of *Sj*Fer0 in *Saccharomyces cerevisiae*. The* fet3fet4 *double mutant strain DEY1453 was transformed with an empty vector pYES2 or with *Sj*Fer0. The wild-type DY1450 strain transformed with an empty vector pYES2 served as a positive control. Samples of 5 μl of a serial dilution (OD_600_ = 1.0, 0.1, 0.01, and 0.001) were spotted onto the induced expression plate with 10 μM FeCl_3_

Compared with the wild-type DY1450 yeast, the growth of the mutant DEY1453 yeast that was transformed with the empty vector (DEY1453-pYES2) was significantly suppressed. Interestingly, the mutant DEY1453 yeast transformed with the recombinant *Sj*Fer0 sequence (DEY1453-pYES2-*Sj*Fer0) was able to rescue the growth of the mutant DEY1453 yeast (Fig. 7).

### RNA-Seq after *Sj*Fer0 interference

To explore how *Sj*Fer0 inhibits the growth and development of schistosome after RNAi, we injected *Sj*Fer0 dsRNA at 14 dpi and 18 dpi and collected worm samples at 22dpi for RNA-Seq.

**Fig. 8:**
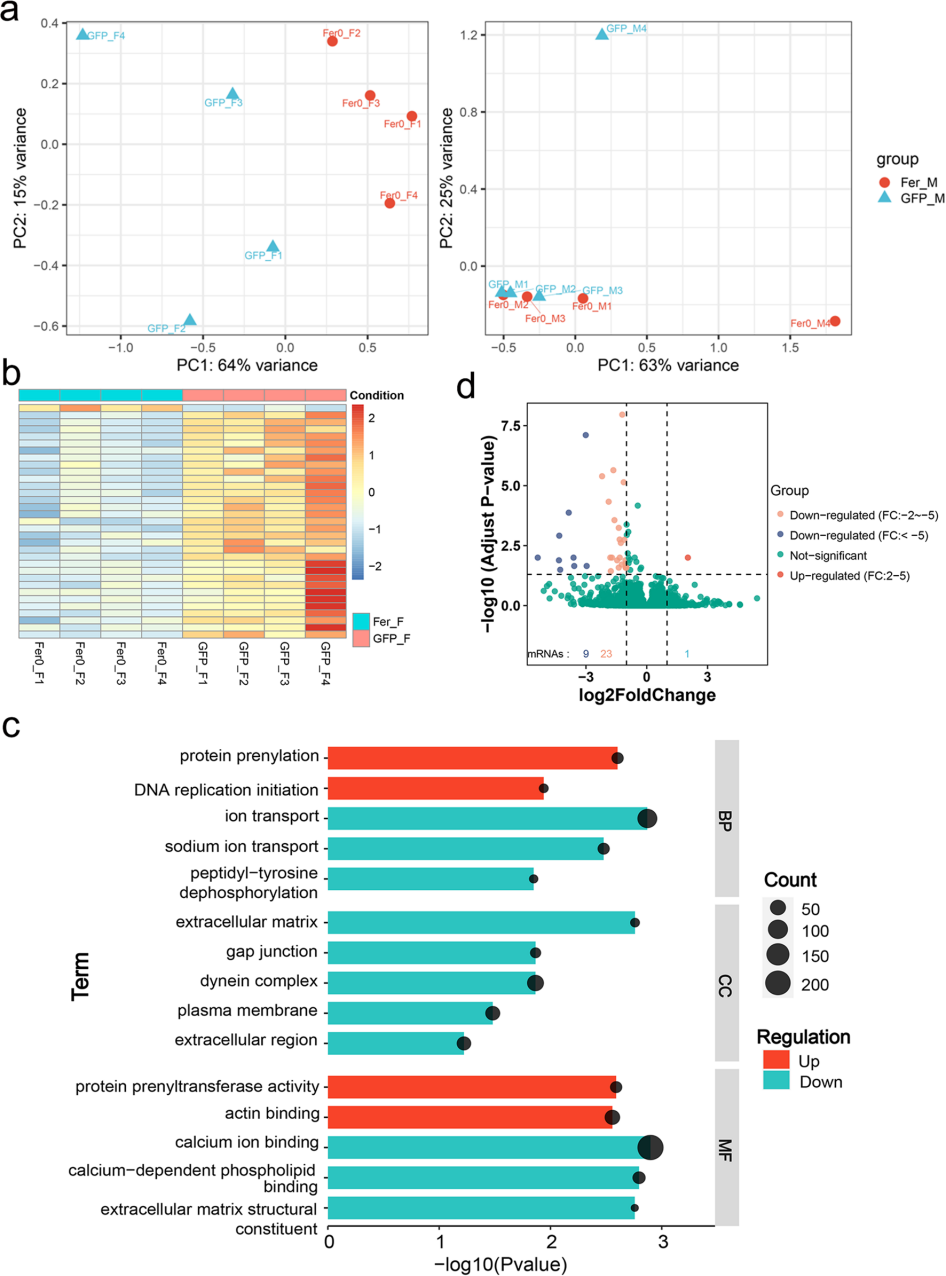
RNA-sequencing after *Sj*Fer0 RNA-interference. Parasites were injected with *Sj*Fer0 and GFP dsRNA at 14 dpi and 18 dpi and collected at 22 dpi. **a** PCA analysis. **b** Heatmap of the female gene expression level. Downregulated and upregulated condition: log2FoldChange; **c** Gene set enrichment analysis. *P* value < 0.05, *q* value < 0.25. **d** Volcano map of differential genes. *P* < 0.05, log2-FoldChange > 1 or log2FoldChange < −1. Abbreviations: BP, Biological process; CC, cellular component; MF, molecular function; PCA, principal component analysis

After splicing the sequencing data and data checking by the QC tool MultiQC, we performed principal component analysis (PCA). We found that the differences between the RNAi group and the control group were more pronounced in females than in males (Fig. 8a). In addition, previous RNAi in vivo experiments showed that *Sj*Fer0 affected growth and development more in females than in males (Fig. 5). Given the poor data quality of males, here we only analyze and discuss the data on females in this section.

**Fig. 9:**
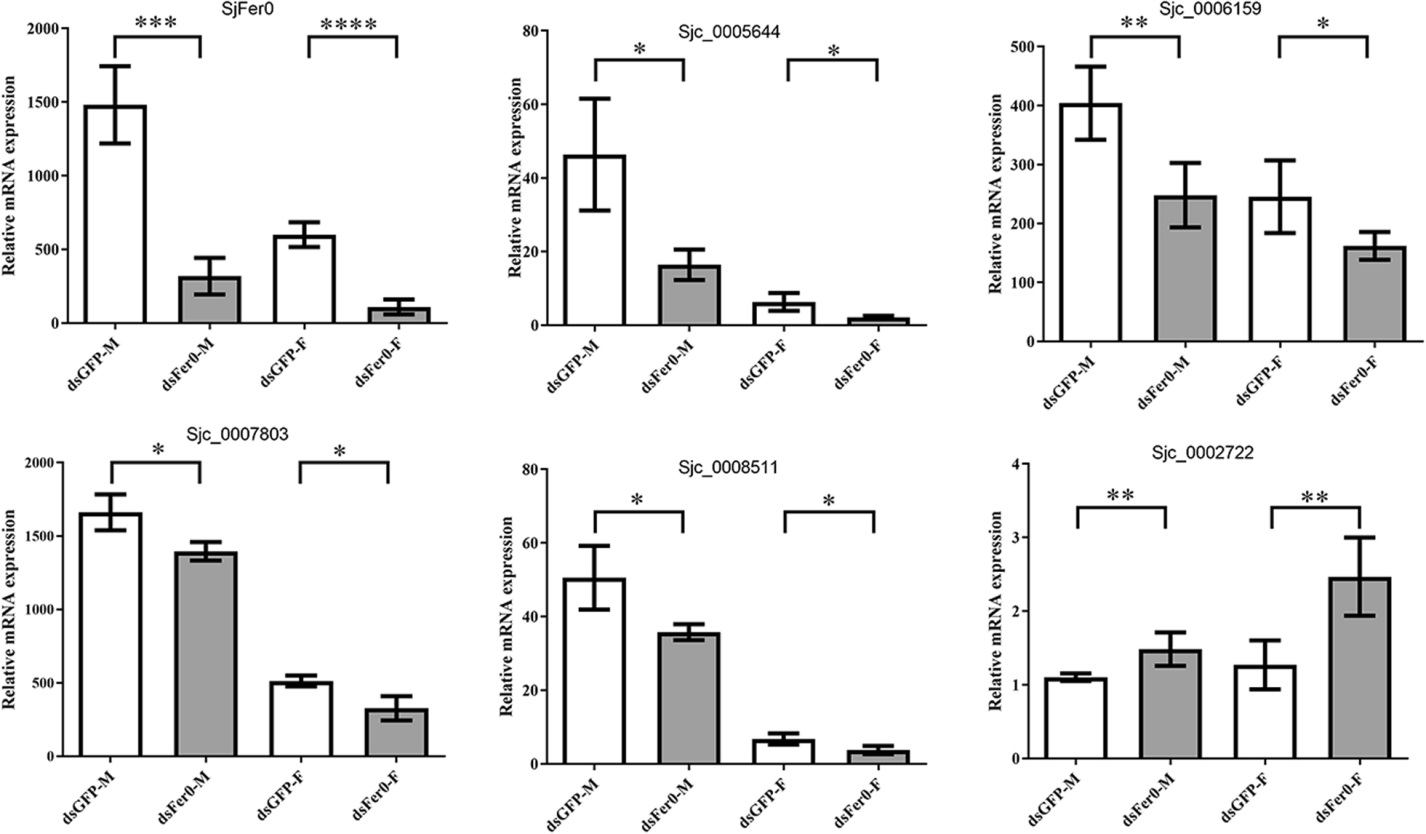
Quantitative PCR verification of representative differential gene expression. The expression changes of our downregulated genes (Sjc_0005644, Sjc_0006159, Sjc_0007803 and Sjc_0008511) and one upregulated gene (Sjc_0002722) were tested by RT-qPCR. Error bars: 95% confidence intervals, *n* = 3. Asterisks indicate signficant difference at *P < 0.05, **P < 0.01, ***P < 0.001 and ****P < 0.0001 (t-test)

The gene expression heatmap showed that most gene expression decreased in females and that only a tiny part increased after *Sj*Fer0 interference (Fig. 8b). The gene set enrichment analysis (GSEA) showed that the ion transport biological process and calcium ion binding molecular function were downregulated and that protein isopentenyl transferase activity molecular function was upregulated (Fig. 8c; Additional file 6: Table S3). Differential expression analysis revealed that one gene was significantly upregulated (log2FoldChange > 1) and 32 were significantly downregulated (log2FoldChange < −1) (Fig. 8d; Additional file 6: Table S3; Additional file 7: Sequences S1). Real-time PCR verified the mRNA levels of the four downregulated genes and one upregulated gene, the levels of which were indeed significantly changed in the *Sj*Fer0 interfering group (Fig. 9; Additional file 8: Table S4).

## Discussion

Thirty-three differential genes were observed after *Sj*Fer0 RNAi. Of these, 32 genes were downregulated and only one gene (Sjc_0002772) was upregulated. Sjc_0002772 expresses a protein that is similar to mouse KIF28P (UniProt entry: D3YXS5; percent identity: 38.9%). The KIF28P gene encodes a microtubule-dependent motor protein required for mitochondrion morphology and the transport of mitochondria in neuronal cells, which is conserved in chimpanzee, Rhesus monkey, dog, cow, mouse, rat, chicken, zebrafish, the nematod* Caenorhabditis elegans* and frog. However, how the upregulation would affect schistosome growth and development is unclear. We selected four downregulated genes to discuss the potential mechanism underlying the impact of *Sj*Fer0 on schistosome growth and development.

Sjc_0005644 and Sjc_0005645 encode cystatin-containing proteins. Cystatin (the cysteine protease inhibitor) is one of the most widely studied host immunomodulatory molecules found in parasites and is primarily secreted by parasites to evade host immune responses [40]. Chen et al. found that a cystatin in *S. japonicum* was a parasite-derived immunosuppressive factor [41]. The Bm-cpi-2 protein of *Brugia malayi* inhibits the presentation of MHC-II-restricted antigens [42]. Therefore, downregulation of *Sj*-cpi-2 may reduce the immune evasion ability of schistosomes and affect growth. In addition, the cystatin cpi-2 protein (Ce-cpi-2; *C. elegans*; UniProt entry: G5ECM9), which is similar to Sjc_0005644 and Sjc_0005645, has been proved to play an essential regulatory role in the process of oogenesis and fertilization [43]. Ce-cpi-2 is required for the uptake and/or processing of yolk proteins during the development of oocytes, probably by regulating the catalytic activity of cysteine proteases cpl-1 and cpz-1. However, whether *Sj*-cpi-2 can regulate the process of oogenesis and fertilization requires further investigation.

The Sjc_0008511 expresses a putative annexin. The annexin protein family binds to cell membrane phospholipids in a Ca^2+^-dependent manner, and different types of annexin proteins may perform various functions [44]. Annexins found in invertebrates, including helminths, are denoted as group B annexins. In *S. mansoni*, annexins are thought to provide structural integrity to the tegument [45]**.** The Sjc_0006159 protein in our study is similar to Drosophila muscle LIM protein (Mlp84B; UniProt Entry: Q24400; percent identity: 47.8%). Mlp84B is a component of muscle sarcomeres [46] [47] and maintains the integrity of muscle tissue; it is essential for normal muscle function of Drosophila [48, 49]. Therefore, *Sj*Fer0 interference may affect muscle formation and schistosome movement.

Sjc_0007803 encodes an insulin-like growth factor-binding protein (IGFBP). IGFBPs [50] exert major effects on cell growth and metabolism. Downregulation of this gene may suppress schistosome growth signal transmission, thereby suppressing worm development.

It has been shown that H-ferritin and L-ferritin mRNA in vertebrates can be regulated at both the transcriptional and post-transcriptional levels [51]. Post-transcriptional regulation is dependent on iron regulatory proteins (IRPs) that bind to the iron-responsive elements (IREs) in the regulatory region of ferritin mRNA [52].* Schistosoma* ferritin mRNA does not have IREs [53], indicating that regulation at the post-transcriptional level is unlikely.* Caenorhabditis elegans* also does not have IREs, and the regulation of the IRPs and ferritin both rely on iron [54]. Nevertheless, *C. elegans* ferritins can be regulated at the transcriptional level through elements called iron-dependent enhancers (IDE). The transcription factor ELT-2 is involved in the regulation of IDE-dependent ferritin and divalent metal transporter 1 (DMT1) mRNA [55]. A homolog of ELT-2 was found in *S. japonicum* (AY809358.1), but whether or not this homolog regulates* Schistosoma* ferritins remains unknown. The hypoxia-inducible factor HIF-1 can inhibit nematode ferritin expression through hypoxia-response elements in the IDE regulatory region during iron deficiency [56]. It would be interesting to explore whether there is a similar regulation mode in schistosomes.

Ferroptosis is a new concept of programmed cell death that has emerged in recent years [57]. Ferritin maintains the homeostasis of iron metabolism [58], and Ras-mutated ferroptosis-sensitive cells express less ferritin than ferroptosis-resistant cells [59], suggesting that ferritins are related to ferroptosis. Thus, it may be interesting to examine whether knockdown of *Sj*Fer0 mRNA will cause cell ferroptosis and thus affect the growth and development of schistosomes.

## Conclusions

The present study found that a new isoform ferritin *Sj*Fer0 of *S. japonicum* affects the growth and development of schistosomula but does not affect egg production of adult female worms. In addition, *Sj*Fer0 can rescue the growth of the* fet3fet4* double mutant *S. cerevisiae* (strain DEY1453), suggesting that it may  promote iron absorption. Finally, RNA-seq was performed after *Sj*Fer0 interference, and it was found that ion transport biological process and calcium ion binding molecular function were downregulated.

## Supplementary Information

**Additional file 1: Table S1.** qPCR primers of *Sj*Fer0, *Sj*Fer1 and *Sj*Fer2.

**Additional file 2: Table S2.** Primers for dsRNA synthesis.

**Additional file 3: Figure S1.** *Sj*Fer1 dsRNA interference in vivo*.*** a** *Sj*Fer1 mRNA expression levels detected by RT-qPCR. Error bars: 95% confidence intervals, *n* = 4. *P < 0.05; ns, no significant difference (*P* > 0.05) (t-test).** b** Worm body length measuring. ns, no significant difference (*P* > 0.05 (t-test), *n* > 30.** c** Schistosome carmine alum staining observed under a fluorescence microscope. Scale bar: 100 um.** d** Schistosome carmine alum staining observed under a laser scanning confocal microscopy (LSCM). Scale bar: 25 um.

**Additional file 4: Figure S2.** *Sj*Fer2 dsRNA interference in vivo*.*** a** *Sj*Fer2 mRNA expression levels detected by RT-qPCR. Error bars: 95% confidence intervals, *n* = 4. *P < 0.05, **P < 0.01 (t-test).** b** Worm body length measurements. *P < 0.05; ns, no significant difference (*P* > 0.05) (t-test), *n* > 30.** c** Schistosome carmine alum staining observed under a fluorescence microscope. Scale bar: 100 um.** d** Schistosome carmine alum staining observed under a laser scanning confocal microscopy (LSCM). Scale bar: 25 um.

**Additional file 5: Figure S3.** Western blot of polyclonal anti-*Sj*Fer0. The main strip is about 24 kDa (*Sj*Fer0). There are non-specific strips, but the concentration is low, indicating that the specificity of polyclonal antibodies is reasonable.

**Additional file 6: Table S3.** Downupregulated and upregulated genes.

**Additional file 7: Sequences S1.** Sequences of the differential expression genes.

**Additional file 8: Table S4.** Differential genes’ qPCR primers.

## Data Availability

All materials and data supporting these findings are contained within the manuscript and supplementary figures.

## Abbreviations

Abcb10: ATP-binding cassette sub-family B member 10
cyts-b561: Cytochromes b561
DAPI: 4’,6-Diamidin-2-phenylindol
DMT1: Divalent metal transporter 1
FPN: Ferroportin
GFP: Green fluorescent protein
HEPH: Hephaestin
NTBI: Non-transferrin-bound iron
PCBP1: Poly (rC) binding protein 1
PSMD4: *26S* Proteasome non-ATPase regulatory subunit 4
Scara5: Scavenger receptor member 5
*Sj*Fer: *S. japonicum* ferritin
Steap3: Six-transmembrane epithelial antigen of prostate 3
Tf: Transferrin
TIM: T cell immunoglobulin-domain and mucin-domain
Tfr1: Transferrin receptor 1
