# Supplementary material for: A new ferritin SjFer0 affecting the growth and development of Schistosoma japonicum
Source: Parasit Vectors. 2022 May 24;15:177. doi: 10.1186/s13071-022-05247-1 (PMC9128280; doi:10.1186/s13071-022-05247-1)
Supplement: Supplementary file 1 — Additional file 1: Table S1. qPCR primers of SjFer0, SjFer1 and SjFer2. [file 13071_2022_5247_MOESM1_ESM.docx]

Table s1. qPCR primers of *Sj*Fer0, *Sj*Fer1, and *Sj*Fer2

| Gene | Sense Forward | Sense Reverse |
| --- | --- | --- |
| *Sj*Fer0 | 5’-GAATTACATAAGGCAGCATCG-3’ | 5’-TTCATCTGTTCTCGGAGGA-3’ |
| *Sj*Fer1 | 5’-GCATCGGGATACACACTTT-3’ | 5’-CCACACGGTTGAGATTCG-3’ |
| *Sj*Fer2 | 5’-TGATCCAGCATTGACAGATT-3’ | 5’-CCAACACGTTGTGTTTCTG-3’ |
| PSMD | 5’-CCTCACCAACAATTTCCACATCT-3’ | 5’-GATCACTTATAGCCTTGCGAACAT-3’ |
