# Supplementary material for: A new ferritin SjFer0 affecting the growth and development of Schistosoma japonicum
Source: Parasit Vectors. 2022 May 24;15:177. doi: 10.1186/s13071-022-05247-1 (PMC9128280; doi:10.1186/s13071-022-05247-1)
Supplement: Supplementary file 2 — Additional file 2: Table S2. Primers for dsRNA synthesis. [file 13071_2022_5247_MOESM2_ESM.docx]

Table s2. Primers for dsRNA synthesis

| Gene | Sense Forward | Sense Reverse |
| --- | --- | --- |
| GFP | 5’-TAATACGACTCACTATAGGGAGAAGTCAGTGGAGAGGGTGAAG-3’ | 5’-TAATACGACTCACTATAGGGAGAACTAGTTGAACGGATCCATC-3’ |
| *Sj*Fer0 | 5’-TAATACGACTCACTATAGGGAGAATAACGGTGAGGTCAGAGAT-3’ | 5’-TAATACGACTCACTATAGGGAGAATTGAACTCCATCGGTACAC-3’ |
| *Sj*Fer1 | 5’-TAATACGACTCACTATAGGGAGAATGAAGAATGTGAAGCTGGTA-3’ | 5’-TAATACGACTCACTATAGGGAGAAATGGCATGGAGACCTGAA-3’ |
| *Sj*Fer2 | 5’-TAATACGACTCACTATAGGGAGAACGCCAAAGAATGTGAAGAT-3’ | 5’-TAATACGACTCACTATAGGGAGAAGCATCCTCTAATCCATTGAA-3’ |
