# Supplementary figures and images for: A new ferritin SjFer0 affecting the growth and development of Schistosoma japonicum
Source: Parasit Vectors. 2022 May 24;15:177. doi: 10.1186/s13071-022-05247-1 (PMC9128280; doi:10.1186/s13071-022-05247-1)

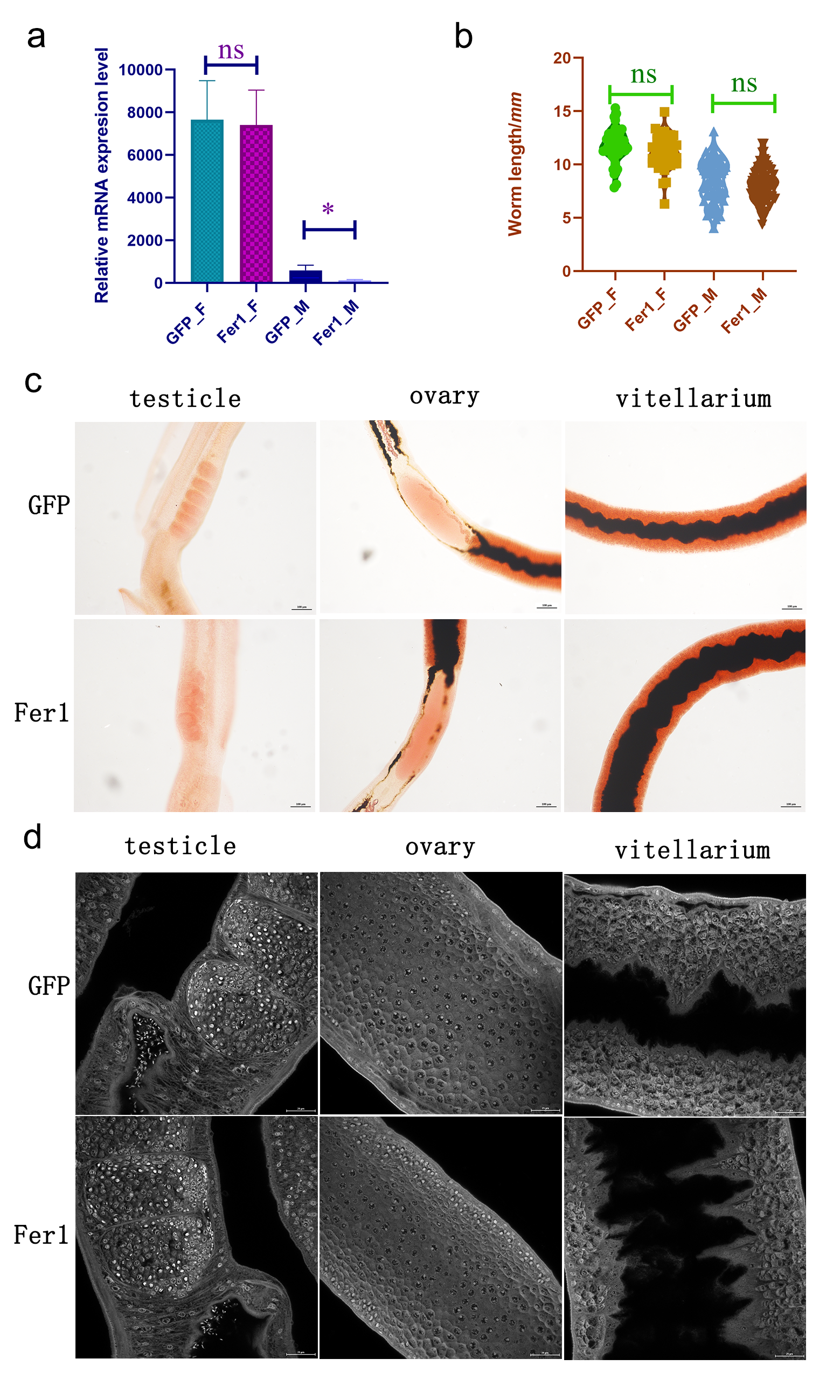

Supplement: Supplementary file 3 — Additional file 3: Figure S1. SjFer1 dsRNA interference in vivo. a SjFer1 mRNA expression levels detected by RT-qPCR. Error bars: 95% confidence intervals, n = 4. *P < 0.05; ns, no significant difference (P > 0.05) (t-test). b Worm body length measuring. ns, no significant difference (P > 0.05 (t-test), n > 30. c Schistosome carmine alum staining observed under a fluorescence microscope. Scale bar: 100 um. d Schistosome carmine alum staining observed under a laser scanning confocal microscopy (LSCM). Scale bar: 25 um. [file 13071_2022_5247_MOESM3_ESM.tif]

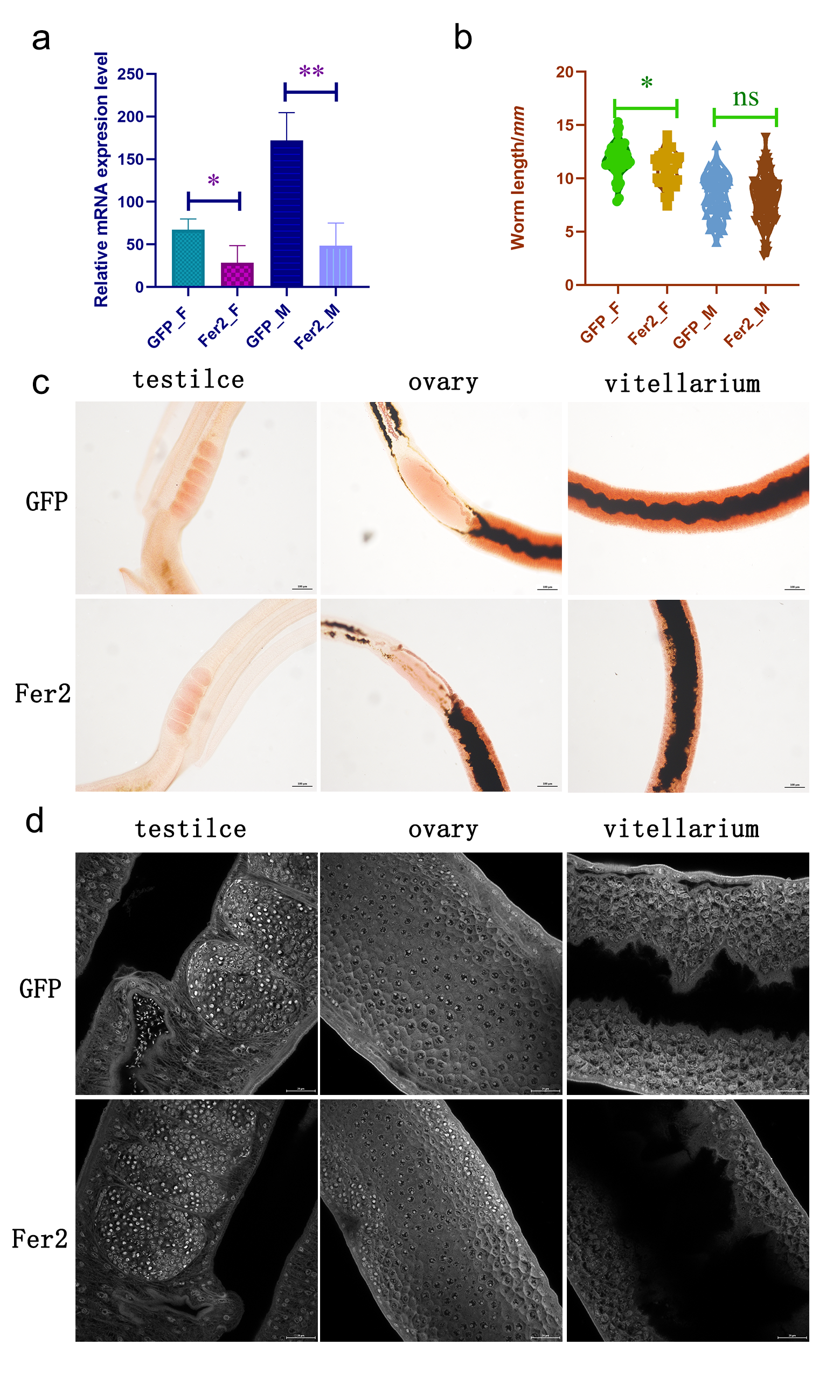

Supplement: Supplementary file 4 — Additional file 4: Figure S2. SjFer2 dsRNA interference in vivo. a SjFer2 mRNA expression levels detected by RT-qPCR. Error bars: 95% confidence intervals, n = 4. *P < 0.05, **P < 0.01 (t-test). b Worm body length measurements. *P < 0.05; ns, no significant difference (P > 0.05) (t-test), n > 30. c Schistosome carmine alum staining observed under a fluorescence microscope. Scale bar: 100 um. d Schistosome carmine alum staining observed under a laser scanning confocal microscopy (LSCM). Scale bar: 25 um. [file 13071_2022_5247_MOESM4_ESM.tif]

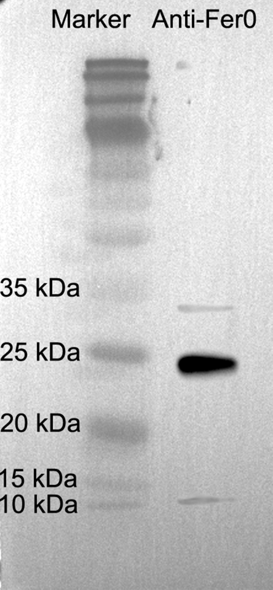

Supplement: Supplementary file 5 — Additional file 5: Figure S3. Western blot of polyclonal anti-SjFer0. The main strip is about 24 kDa (SjFer0). There are non-specific strips, but the concentration is low, indicating that the specificity of polyclonal antibodies is reasonable. [file 13071_2022_5247_MOESM5_ESM.tif]
